# Pharmaceutical company promotional payments to English general practices: a longitudinal study

**DOI:** 10.3399/BJGPO.2024.0281

**Published:** 2026-01-14

**Authors:** Shai Mulinari, Minahil Malik, James Larkin, Mostafa Elsharkawy, Tom Fahey, Frank Moriarty, Piotr Ozieranski

**Affiliations:** 1 Department of Sociology, Lund University, Lund, Sweden; 2 Department of General Practice, RCSI University of Medicine and Health Sciences, Dublin, Ireland; 3 Member, Cairo, Egypt; 4 School of Pharmacy and Biomolecular Sciences, RCSI University of Medicine and Health Sciences, Dublin, Ireland; 5 Department of Social and Policy Sciences, University of Bath, Bath, UK

**Keywords:** general practice, pharmaceutical companies, marketing, longitudinal studies

## Abstract

**Background:**

General practices have been a long-standing focus of pharmaceutical promotion, but their financial relationships with pharmaceutical companies remain understudied.

**Aim:**

To examine pharmaceutical company payments to general practices in England from 2015–2022, focusing on changing patterns of payments and what this reveals about companies’ marketing.

**Design & setting:**

Descriptive analysis of pharmaceutical company payments made to practices using data from industry’s Disclosure UK database, covering 4430 recipient practices and 54 companies over an 8-year period.

**Method:**

Annual Disclosure UK data from 2015–2022 were merged, identifying practices using a novel algorithm-based methodology, and categorising payments by type (for example, donations and grants, event sponsorship). Trends were analysed by company and payment type. The Gini coefficient measured payment concentration, and the persistence of relationships was assessed over time.

**Results:**

Pharmaceutical payments to general practices rose from £2.5 million in 2015 to £7.5 million in 2022. While 54 companies made payments, just one company, Chiesi — marketing commonly prescribed respiratory inhalers — accounted for more than 50% of the payment value from 2017 onwards. More than 40% of practices received payments from only one company, and 74% of company-practice relationships lasted just 1 study year. A few companies dominated, with a Gini coefficient of 0.86, driven by Chiesi’s payments.

**Conclusion:**

The growing scale and concentration of payments and the dominance of one company raises concerns about bias in general practice. Future research should investigate the impact of payments on clinical decision making, but to do so, payment disclosures need enhanced transparency, particularly through including product-specific payment details.

## How this fits in

Pharmaceutical companies have historically focused on influencing individual doctors’ prescribing, but recent evidence suggests a shift towards targeting NHS organisations. While financial relationships between drug companies and general practices are acknowledged, their extent and patterns remain unclear. This research reveals a major increase in drug company promotional payments to general practices in England, largely driven by major contributions from one company, Chiesi, which markets respiratory inhalers commonly prescribed by GPs. These findings highlight the potential for undue influence on prescribing decisions, stressing the need for GPs to be vigilant about industry influence and the importance of enhanced transparency in the reporting of company payments to general practices and their staff.

## Introduction

General practices have long been the focus of pharmaceutical promotion, yet their interactions with drug companies remain poorly understood. Over 20 years ago, research showed that pharmaceutical sales representatives influenced GPs’ prescribing, sometimes leading to the use of more expensive or less effective drugs.^1,2^ Recent studies suggest a shift in focus, with companies increasingly targeting NHS organisations rather than individual doctors.^3,4^ This change reflects the growing impact of organisational priorities on prescribing.^4^ Accordingly, the Independent Medicines and Medical Devices Safety Review (IMMDS), chaired by Baroness Julia Cumberlege, describing prominent cases of avoidable patient harm in the NHS, highlighted concerns about institutional conflicts of interest arising from industry’s promotion, beyond those of individual doctors.^5^

Historically, no data were available on how pharmaceutical companies engaged with GP practices for promotional purposes. This changed with the 2015 launch of the Disclosure UK database, managed by the Association of the British Pharmaceutical Industry (ABPI). Disclosure UK reports drug companies’ payments to healthcare professionals^3^ and healthcare organisations^6^ (HCOs), categorised according to predefined criteria. Supplementary Text 1 provides detailed definitions of the most relevant reported payment categories for GP practices: donations and grants, event sponsorship, and joint working.

Despite this progress, isolating payments to GP practices remains challenging. Disclosure UK does not categorise recipients by type^7^ and its inconsistent recipient naming and location data further complicate analysis.^3^ Additionally, the database provides limited transparency regarding who ultimately receives the funds (for example, practices may receive certain payments on behalf of their GPs), and how they are used. The only study of English GP practices using Disclosure UK data found that in 2015, they received 6.5% of all promotional payments made to HCOs, which amounted to £2.6 million across 1643 practices from 34 companies.^8^ Bayer was the largest donor, responsible for nearly 30% of these payments.

However, it remains unclear whether companies establish long-term financial relationships with GP practices, and whether practices typically receive payments from many companies or a select few. Both long-term and ’exclusive’ ties are associated with a higher risk of bias.^9–11^ It is also uncertain whether the dominance of a single company, as seen in 2015, was an anomaly or reflects a trend. We also know little about whether examining specific companies’ payment patterns could offer insights into their marketing, which is largely because Disclosure UK lacks information on products marketed through the payments.^8^

This study addresses these gaps by examining patterns of pharmaceutical industry payments to GP practices in England since 2015, using an algorithm developed to identify payments to practices reported in Disclosure UK.

## Method

### Data source: Disclosure UK

We analysed Disclosure UK datasets from 2015–2022, downloaded from the ABPI website^12^ around the time of release each year. Disclosure UK has been described in detail elsewhere.^3,6,13^ At the time of analysis, the most recent available dataset was for 2022. Each dataset includes payment data reported by companies subscribing to the ABPI Code of Practice. This includes approximately 175 companies, which together supply the vast majority of branded medicines used by the NHS, according to the ABPI.^12^

### Data processing and integration

The eight datasets were appended together, retaining recipients labelled as HCOs. Since payments related to company research and development (R&D) are reported without named recipients^14^ — as allowed under the ABPI Code of Practice^13^ — we exclude these payments, focusing instead on non-R&D payments, or promotional payments, which must include this information.^13^ Promotional payments are categorised in the database as either ‘donations (physical items, services, or benefits-in-kind) and grants (funding)’, ‘event sponsorship (for example, HCO-organised lunchtime presentations in GP practices, management training courses, patient support group meetings)’, ’event registration fees’, ‘event travel and accommodation (for example, payments for GP practice staff registration, travel, and accommodation at events or meetings when staff identities these are “not known to the company”)’, ‘joint working (where HCOs and drug companies pool resources)’, ‘fees for service and consultancy (for example, chairing and speaking at meetings, assistance with training and participation in advisory boards, received by HCOs on behalf of their employees)’, or ’expenses for service and consultancy’.^8^

To account for between-company and over-time differences in reporting,^13^ we aggregated each company’s annual payments to individual recipients, such as GP practices by payment category (for example, donations and grants). All payment values are presented in 2022 GBP, adjusted for inflation using the Bank of England’s inflation calculator.^15^

### Identification of GP practices in Disclosure UK

**Figure 1.**
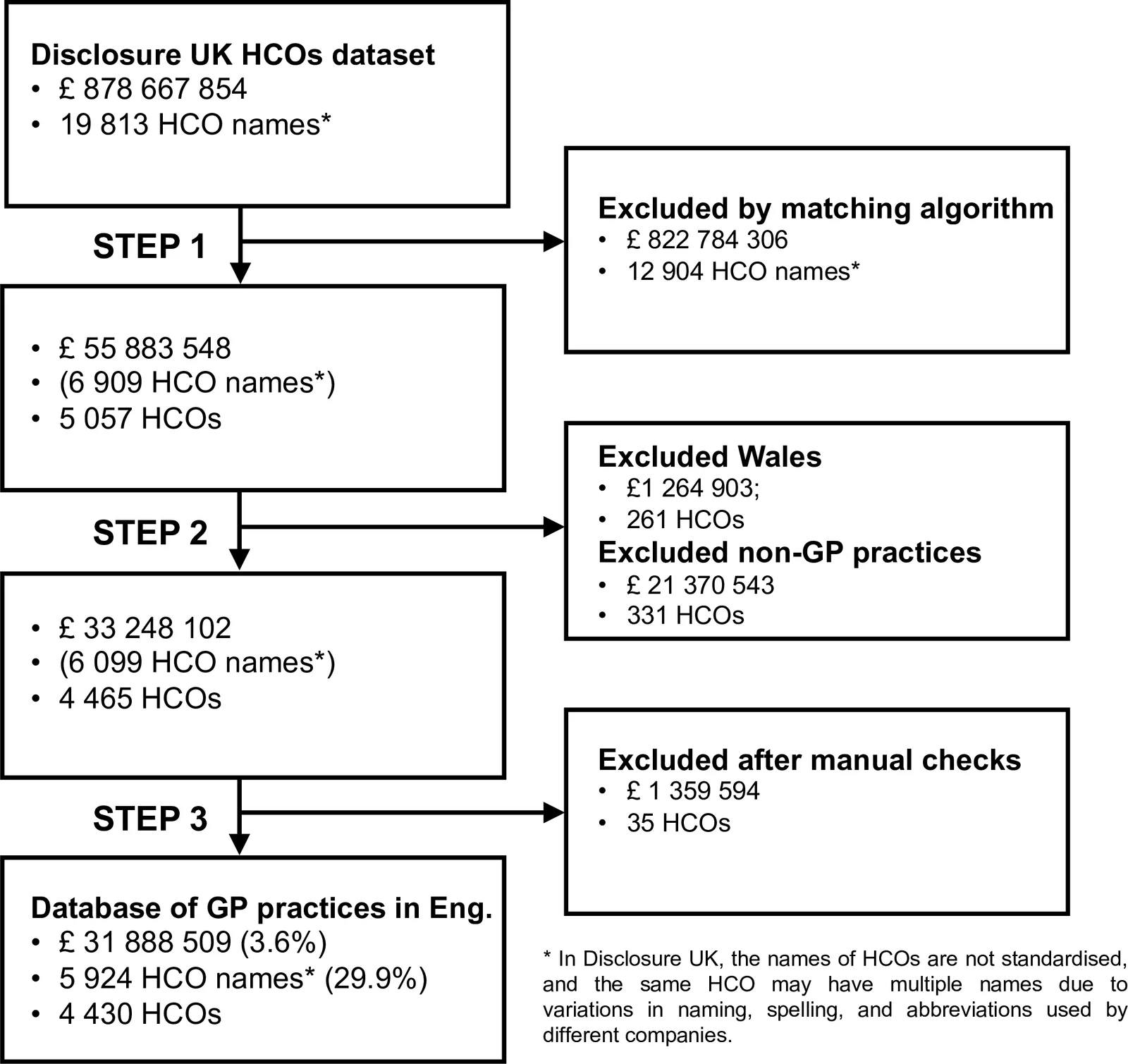
Identification of 4430 English GP practices in Disclosure UK, 2015–2022. HCO = healthcare organisation

The combined dataset (2015–2022) included payments to HCO recipients with 19 813 unique names, totalling £878.7 million (Figure 1). HCO names in Disclosure UK are not standardised, which may result in multiple names for the same HCO.^8^

Figure 1 outlines the approach used to identify English GP practices.

#### Step 1

We developed a matching algorithm to identify potential practices by comparing HCO ’names’, ‘addresses’, ‘locations’, and ‘postcodes’ reported by companies in Disclosure UK with similar information about prescribing primary care HCOs in the ‘epraccur’ file (November 2023) from NHS England.^16^ The matching algorithm classified an HCO as a potential GP practice if its postcode in Disclosure UK matched an organisation’s postcode in the epraccur file, and if it met at least two additional matching criteria for name, location, or address information across the two datasets. Details are in Supplementary Text 2; the code is available on GitHub (https://github.com/MostafaElshark/GP_Project). This step excluded 12 904 HCO names, accounting for £822.8 million. The remaining 6909 HCO names were matched to 5057 unique primary care HCOs, each with a unique six-character organisation code in the epraccur file. These primary care HCOs included GP practices but also, for example, public health, hospital services, and cancer screening units.

#### Step 2

We excluded primary care HCOs in Wales (261 recipients; £1.26 million) and non-practices with primary care organisation codes (331 recipients; £21.37 million). Welsh organisation codes start with ‘W.’ Each HCO’s classification as a GP practice was confirmed using the epraccur file (November 2023) from NHS England.^16^ The file contains a variable indicating whether an organisation is a GP practice.

#### Step 3

We manually confirmed remaining entries as GP practices to address potential inaccuracies in naming, address, and location data in Disclosure UK.^8^ Supplementary Text 3 details this process. This step excluded an additional 165 HCOs, who received £1.36 million.

The final dataset included 4430 GP practices, receiving payments worth £31.9 million (Figure 1).

### Analysis

We analysed payment trends descriptively, categorising the data by year, category, company, and the recipient GP practice. Sums, medians, and quartiles were calculated, and scatter plots, box plots, jitter plots were used to visualise distributions.

To assess payment concentration among donor companies and recipient practices, we calculated Gini coefficients for the value of payments, which ranges from 0 (perfect equality, where all companies or practices provide or receive equal amounts) to 1 (maximum inequality, where one company or practice provides or receives all payments).^3^

To gain insight into company-specific patterns, we compared the payment trends of the 10 companies with the highest cumulative payments over the entire period. We noted the major dominance by a single company, Chiesi — specialising in the respiratory inhaler market — and therefore separately examined its payments compared with all other companies and to competitors also marketing single-inhaler triple therapies combining an inhaled corticosteroid (ICS), a long-acting β2-agonist (LABA), and a long-acting muscarinic antagonist (LAMA).

We analysed the exclusivity of company-practice relationships by calculating the percentage of practices receiving payments from either one or multiple companies, and visualised company overlaps using Venn diagrams. Additionally, to assess the persistence of financial relationships (that is, whether practices continued receiving payments from the same company over time), we calculated the proportion of recurring payments across multiple years. Analyses were done in RStudio (version 2024.04.2).

## Results

### Overview of payments to GP practices

Between 2015 and 2022, companies reported £31.9 million in payments to GP practices in England. These payments were made by 54 companies, averaging 30 companies annually, and received by 4430 practices, averaging 1363 practices annually. As Figure 1 shows, these payments accounted for 3.6% of promotional payments to HCOs in Disclosure UK, but, notably, 29.9% (*n* = 5924) of HCO names in the database, highlighting that while industry pays relatively little to practices compared with other HCOs, industry-practice relationships are widespread. Most of the money was categorised as ’donations and grants’ (87.1%; £27.8 million). ’Event sponsorships’ comprised 6% (£2.1 million), and ’joint working’ comprised 5.4% (£1.7 million). Together, these categories represented 99.1% of the total payments.

### Trends in payments

**Figure 2.**
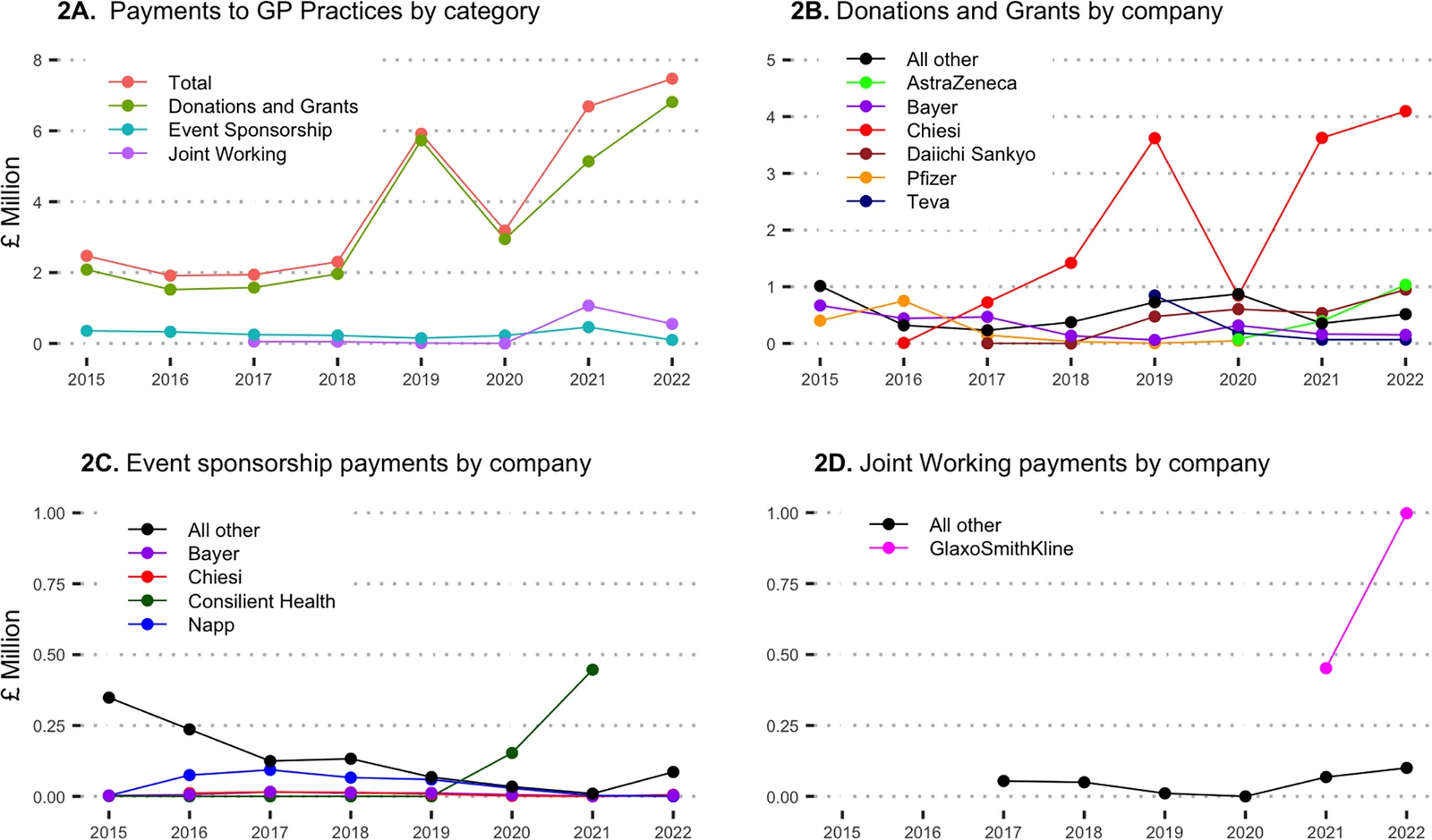
Trends in pharmaceutical industry payments to GP practices in England, 2015–2022, by category and company

Figure 2A demonstrates that annual payments remained steady from 2015 to 2018 (£1.9–2.5 million) but increased in 2019 to £5.9 million, driven largely by ’donations and grants’. This category increased from £2.0 million in 2018 to £6.8 million in 2022. Although ‘joint working’ payments increased after 2020, their share remained small (7.4% in 2022), with ‘donations and grants’ comprising 91.2% in 2022. A temporary dip in total payments occurred in 2020, with payments rebounding subsequently, and reaching £7.5 million in 2022 (see also Table 1). The 2020 dip was linked to smaller annual payments per practice, evidenced by the drop in the median and upper quartile values (Table 1).

**Table 1.** Patterns in payments to GP practices in England, 2015–2022

Year	Sum, £	Practices	Companies	Median (IQR), £	Minimum, £	Maximum, £	Gini^a^ for companies	Gini^a^ for practices
**2015**	2 470 787	1315	32	530 (250–1220)	11.7	36 600	0.79	0.66
**2016**	1 913 949	1176	35	290 (212–789)	6.2	121 000	0.84	0.72
**2017**	1 940 319	1248	34	354 (212–944)	10.4	63 720	0.83	0.66
**2018**	2 302 502	911	28	421 (230–1574)	11.5	58 847	0.84	0.66
**2019**	5 916 306	1279	34	1300 (283–4520)	0	31 075	0.90	0.56
**2020**	3 184 613	1288	34	790 (336–1877)	1.1	89 060	0.72	0.61
**2021**	6 689 699	1709	24	627 (330–2330)	13.6	176 329	0.84	0.64
**2022**	7 470 334	1981	27	951(420–2693)	16	108 957	0.86	0.62
**Total**	31 888 508	**4430**	**54**	**632 (256–1870)**	**0**	176 329	0.86	0.64

Values are presented in 2022 GBP. ^a^Gini coefficients measure payment concentration among company donors and practice recipients from 0 to 1. IQR = interquartile range

### Differences between companies

There was a high concentration of payments, particularly among companies. The overall Gini coefficient was 0.86 for companies, peaking at 0.90 in 2019 (Table 1). This large concentration reflects that one company, Chiesi, accounted for 45.2% of total payments (£14.4 million), while the top 10 donors contributed 88.2% (£28.1 million) (Supplementary Table 1). Payment strategies varied among top donors. For example, 99.5% of Chiesi’s payments were ‘donations and grants’, GSK prioritised ‘joint working’ (70.2%), and Consilient Health prioritised ‘event sponsorships’ (64.6%).

Company funding patterns also shifted over time. Bayer and Pfizer dominated in 2015 and 2016 with large ‘donations and grants’ contributions, but their investments diminished later, while Chiesi’s contributions grew rapidly (Figure 2B). Daiichi Sankyo and AstraZeneca also increased their ‘donations and grants’ in recent years, although still far behind Chiesi, which accounted for 51.6% of total ‘donations and grants’ and nearly 60% between 2017 and 2022. Consilient Health’s ‘event sponsorships’ were concentrated in 2020–2021 (Figure 2C), while GSK’s ‘joint working’ payments were concentrated in 2021–2022 (Figure 2D).

**Figure 3.**
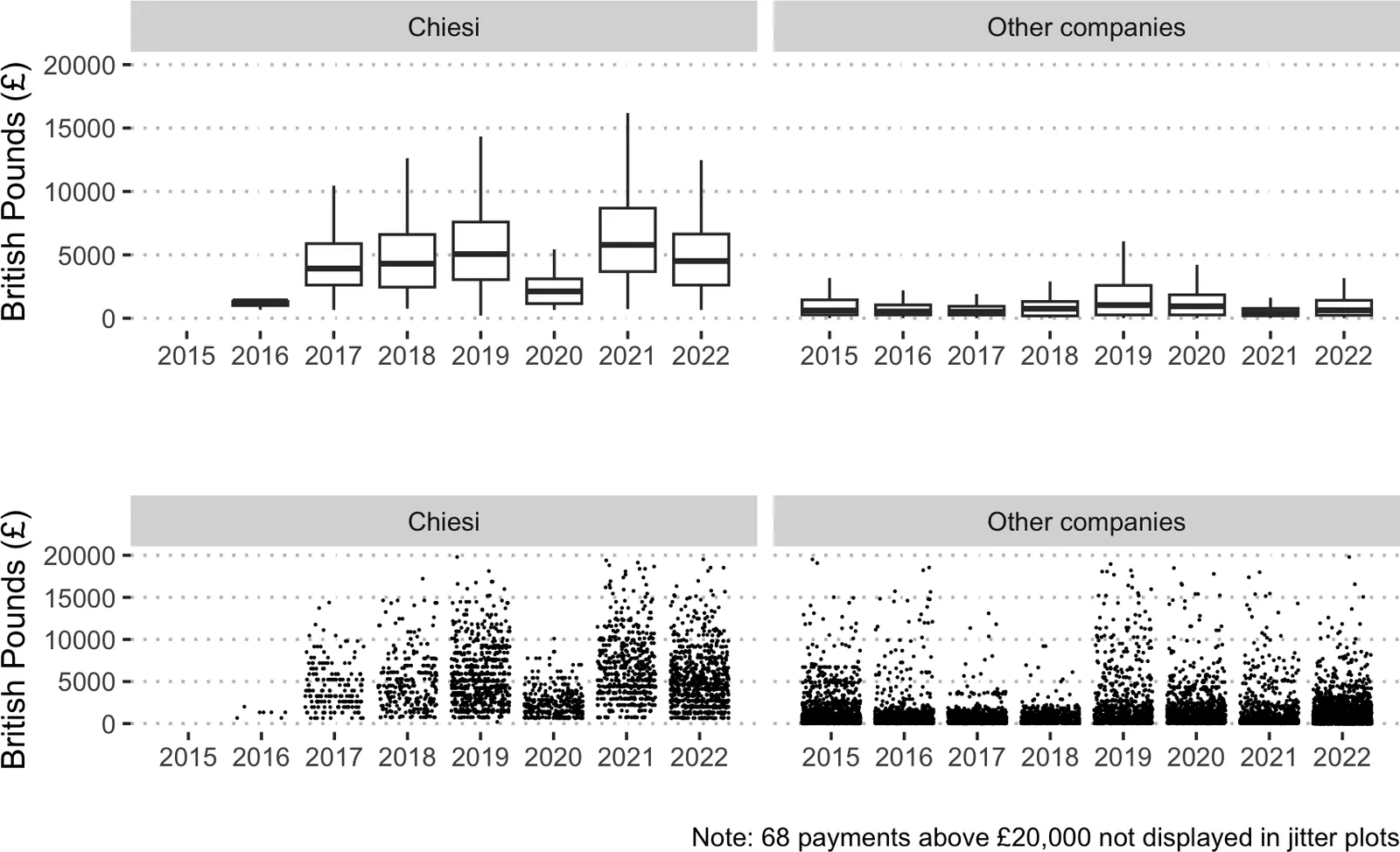
Donations and grants to GP practices in England, 2015–2022: Chiesi compared with all other companies

Chiesi’s contributions since 2017 drove the overall increase in payments for the studied period (Figure 2A–B). Similarly, Chiesi’s temporary drop in payments in 2020, from £3.6 million to £0.8 million, were behind the drop in total values. This 76.7% drop reflected fewer practices receiving ‘donations and grants’ payments (620 to 347) and lower amounts: the median dropped by 58.2% (£5060 to £2117) (Figure 3). However, Chiesi’s ‘donations and grants’ payments rebounded to £3.6 million in 2021 (median £5886) and £4.1 million in 2022 (median £4514), continuing to drive overall trends (Figure 2A and B).

### Chiesi’s payments

Chiesi’s £14.4 million in payments to English GP practices represented 46.6% of its £30.9 million to all UK HCOs over the study period, compared with 4.4% for the other top 10 donor companies combined (£13.7 of £309.9 million), and 2.1% for all the companies in Disclosure UK combined excluding Chiesi (£17.5 of £847.8 million). The comparison of Chiesi’s ‘donations and grants’ with GP practices (£14.3 million) with those from all other companies combined (£13.4 million) (Figure 3) or selected companies (Supplementary Figure 1) shows Chiesi’s distinct relationship with practices. Thus, Chiesi’s ‘donations and grants’ payments to individual practices were substantially larger, with a median of £4414 (interquartile range [IQR]: £2530–£6988) compared with £558 (IQR: £235–£1317) for other companies. Only 0.04% of Chiesi’s ‘donations and grants’ payments were below £650, compared with 54.1% for other companies.

### Exclusivity and persistence in companies’ payments to GP practices

**Figure 4.**
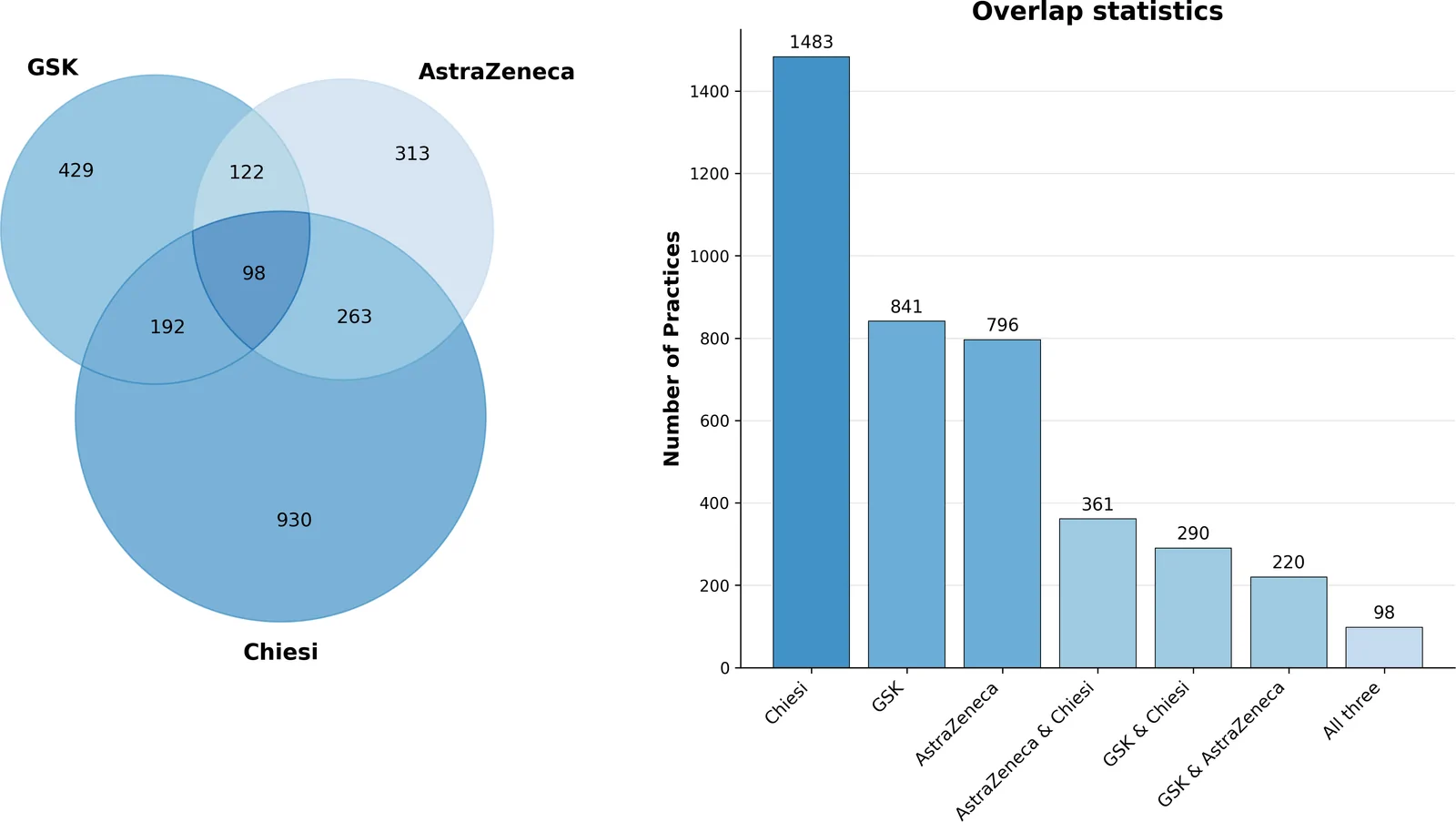
Venn diagram showing limited overlap among GP practices in receipt of payments during the study period from any of the three companies with inhaled corticosteroid (ICS), a long-acting β2-agonist (LABA), or a long-acting muscarinic antagonist (LAMA) single-inhaler triple therapies

The 4430 practices in the database received payments from a mean 2.3 companies over the 8-year period, resulting in 10 285 company-practice relationships (that is, at least one payment from a company to a practice). However, more than 40% of practices received payments from only one company (Table 2). Figure 4 highlights the limited overlap in funding between practices supported by Chiesi, GSK, and AstraZeneca, which are direct competitors, each offering a single-inhaler triple therapy (ICS, LABA, LAMA) for chronic obstructive pulmonary disease (COPD) and/or asthma. Additionally, 74.0% of company-practice financial ties occurred in just 1 year, and 18.3% spanned 2 years (Table 2). In contrast, for Chiesi, these figures were 55.0% and 29.1%, indicating more persistent relationships with practices (Supplementary Table 2).

**Table 2.** Patterns of exclusivity and persistence in payments to GP practices in England, 2015–2022

A. Exclusivity^a^			B. Persistence^b^		
Company funders, *n*	Practices, *n* (%)	Payments,£ (%)	Years payments were made	Company-practice relationships,*n* (%)	Payments,£ (%) for relationships
1	1778 (40.1)	4 919 521 (15.4)	1	7612 (74.0)	14 565 601 (54.0)
2	1161 (26.2)	6 758 268 (21.2)	2	1879 (18.3)	9 583 143 (26.7)
3	660 (14.9)	5 928 728 (18.6)	3	562 (5.5)	3 842 452 (12.0)
4	406 (9.2)	5 446 966 (17.1)	4	157 (1.5)	2 415 978 (4.5)
5	200 (4.5)	2 986 261 (9.3)	5	48 (0.5)	784 209 (1.7)
6	104 (2.3)	2 133 676 (6.7)	6	22 (0.2)	665 129 (0.9)
7	73 (1.6)	2 060 056 (6.5)	7	3 (0.03)	15 819 (0.1)
8	28 (0.6)	774 886 (2.4)	8	2 (0.02)	16 178 (0.1)
9	10 (0.2)	228 673 (0.7)	NA	NA	NA
10	2 (0.05)	111 131 (0.3)	NA	NA	NA
>10	8 (0.2)	540 343 (1.7)	NA	NA	NA
Total practices	4430 (100)	31 888 508 (100)	Total unique relations	10 285 (100)	31 888 508 (100)

Note: Values are presented in 2022 GBP. ^a^Exclusivity indicates the number of companies making payment to a practice over study period. ^b^Persistence indicates the number of years a company made payments to a practice. The values for company-practice relationships exceed the total number of practices because a single practice can receive payments from multiple companies, with each company-practice relation counted separately

## Discussion

### Summary

We found growing financial relationships between GP practices and pharmaceutical companies in England, with promotional payments rising from £2.5 million in 2015 to £7.5 million in 2022. However, given that GP practices received an average of £1.49 million each from the NHS in 2021–2022 to fund their day-to-day activities,^17^ industry payments are unlikely to be a major source of income relative to funding obtained from the NHS. Instead, these funds likely focus on specific activities and events.

Chiesi, a relatively small company^18^ active mainly in the respiratory disease market,^19^ overtook Bayer’s dominant position reported for 2015.^8^ Chiesi accounted for more than 50% of payments from 2017–2022, primarily through donations and grants, driving the overall trends. Of Chiesi’s UK-wide promotional payments to HCOs, 46.6% went to English practices, compared with just 4.4% for the other top 10 drug company contributors to GP practices combined, reflecting its strategic focus on practices. Notably, Chiesi’s payments to individual practices were distinctively larger, as demonstrated by the much larger median value of its payments, raising concerns about their influence on prescribing.

We also examined the exclusivity and persistence of company-practice relations. More than 40% of included practices received payments from only one company, indicating high exclusivity, although 74.0% of company-practice relations lasted just one study year, and 18.3% spanned 2 years, suggesting that while exclusive, many relations are short-lived.

### Strengths and limitations

We developed a robust, novel methodology for identifying GP practices in Disclosure UK. However, the study relies on self-reported data from companies, which may be incomplete or inaccurate.^20,21^ Lack of product-specific payment information^22^ and description of funded activities further limits the analysis.^6,23^ US research has shown a positive correlation between payments to hospitals and affiliated physicians,^24^ which may also apply to practices and employed GPs, and which could enhance the observed patters. However, Disclosure UK does not include organisation and employer codes, preventing linking individual GPs to the practices where they work.

### Comparison with existing literature

This study adds to existing UK research by bringing a closer focus on institutional conflicts of interest,^3,8,12,25–27^ which has been highlighted by the IMMDS review,^5^ but is largely missing from US research, which only covers payments to individual physicians and teaching hospitals.^28^ Although concentration of payments among a few companies is a consistent pattern in the UK,^3,8,25^ Chiesi’s dominance in relation to English practices is unprecedented, particularly given its smaller size (often not ranking among the top 50 pharmaceutical companies based on global prescription drug sales)^18^ and limited product portfolio. Comparable patterns were seen in Denmark, where AbbVie accounted for 23% of payments to patient organisations,^9^ but AbbVie is a top-ranking company in terms of prescription drug sales.^18^

Chiesi’s key products for GP practices are inhalers: Fostair (ICS or LABA) and more recently Trimbow (ICS, LABA, or LAMA), launched in 2017 for COPD treatment.^19^ Payments to practices surged from 2017, which coincided with Trimbow’s market launch. However, without product-specific details of payments, the connection to Trimbow’s market launch cannot be confirmed using Disclosure UK. GSK and AstraZeneca have comparable ICS, LABA, and LAMA inhalers.^29^ Notably, AstraZeneca’s payments rose markedly after the launch of its triple therapy Trixeo in 2020, but we found limited overlap in the practices funded by Chiesi, AstraZeneca, and GSK, suggesting companies focus payments on specific practice segments.

We also found contrasting payment strategies among companies. For example, while Bayer and Chiesi engaged a similar number of practices, the total and average values differed substantially: £14.3 million for Chiesi (median = £4414) versus £2.4 million for Bayer (median = £530). This indicates Chiesi follows a ‘high-value’ payment strategy, while Bayer opts for a ‘high-frequency, low-value’ approach.^8^ While the pandemic may have briefly reduced Chiesi’s high-value payments in 2020, they surged again in 2021, possibly linked to Trimbow’s expanded approval for asthma in 2021.^19^

### Implications for research and practice

This study underscores the need to investigate the potential impact of industry payments on GP prescribing in the UK, similar to research conducted in the US particularly concerning single-inhaler triple therapies, which may increase the risk of ICS-induced pneumonia.^30,31^ US studies have shown a link between manufacturer payments and increased prescribing of their inhalers.^32^ From a policy perspective, improving the information provided in payment disclosures is essential, by adding product-specific payment details, organisation and employer codes, and descriptions of funded activities. This would provide insights into the role of industry payments in clinical decisions and help mitigate conflicts of interest in health care.

## Data

The dataset relied on in this article is available from the corresponding author on reasonable request.
